# *In situ* veritas: combining omics and multiplex imaging can facilitate the detection and characterization of cell-cell interactions in tissues

**DOI:** 10.3389/fmed.2023.1155057

**Published:** 2023-06-01

**Authors:** Uwe Ritter

**Affiliations:** ^1^Chair for Immunology, University of Regensburg, Regensburg, Germany; ^2^Department for Immunology, Leibniz Institute for Immunotherapy (LIT), Regensburg, Germany

**Keywords:** proteomics, RNA-seq, spatial transcriptomics, multiplex imaging, physically interacting cells, dendritic cells, T cells

## Immunity—The consequence of coordinated cellular interactions

In 1898, microscopic examinations were used to study changes in lymphoid tissues and cell distribution in pathological settings (1). More than 100 years after these first discoveries, the complexity of lymphoid organ composition and the existence of distinct immune-cell subpopulations with a diverse set of functions has been described.

For the adaptive immune system to function efficiently, complex series of spatial and temporal interactions between specialized immune cells must take place. This has been a field of vigorous interest, exemplified by the fact that nearly 100.000 papers have been published dealing with the keywords “interaction” and “immune cells” (PubMed search March 2023). Understanding cell-cell communication—resulting in immunological pathways—is being supported by a range of sophisticated analysis pipelines, ranging from *in vitro* and *ex vivo* single-cell sequencing analysis to genic analysis of immunological alterations. In this context, it is commonly accepted that cells can communicate through juxtacrine and paracrine processes (2). Signal transmission and reception between neighboring cells is fundamentally involved in the regulation of immunological processes, ranging from tissue homeostasis to defense mechanisms against tumour cells or pathogens. However, the final decryption of immunological programs responsible for coordinated and dynamic immunological adaption is multi-factorial and remains challenging.

## Exploring the unknown below the surface of tissues—From single cell omics to spatial transcriptomics

Flow-cytometric analysis represents a central pilar of immunophenotyping (3). Based on this technique, it could be shown that immune cells sense and release many molecular mediators capable of modifying immune cell development, phenotype, and function. However, the limited availability of fluorescently labeled antibodies imposes limitations on the detection of different epitopes. Consequently, other approaches such as single-cell RNA sequencing (scRNA-seq) have been established, allowing an upscaling of the analytic dimensions (4). What was previously impossible becomes routine. Transcriptomic datasets in combination with computational analytic pipelines can match raw data with cell clusters of interest and identify biomarkers involved in the developmental trajectory of immune cells (5–7). While these sequencing approaches improve our ability to analyse different cell populations in a variety of contexts, some limitations remain (5). Especially the enzymatic digestion of tissues is critical, as mediators released during tissue processing for single-cell analysis can result in cell activation or death (6). Consequently, rare cell populations can be lost and valuable information about cell-cell interactions and the transcriptome are overlooked. Most importantly, single-cell preparations abolish the spatial context of the cell populations and information about cell-cell interactions become inaccessible (2). Spatial transcriptomics (ST) (7, 8) address these shortcomings by detecting and localizing mRNA transcripts within tissues (2) and became “Method of the Year” in 2021 (9).

In contrast to early *in situ* hybridization techniques which only detect a single transcript (10), ST can detect a broad range of genes expressed within defined detection spots, containing barcoded poly-T oligonucleotides capable of trapping their complementary tissue mRNA. However, the resolution of detection spots can range from two to 55 microns [2 μm: High-definition spatial transcriptomics (11), 10 μm Slide-seq, Slide-seq v2 (12, 13); 55 μm: Visium spatial gene Expression (7)], leading to considerable uncertainty regarding the precise cell assignment of any identified mRNA. These relatively large spot areas represent therefore a major limitation (14) to this technique, since it is challenging to assign the generated RNAseq data to a distinct “cell type” within a sampled portion of tissue. Furthermore, even with a tissue thickness of less than seven microns, the processed tissue-sample still represents a three-dimensional cell layer (15, 16) containing cells stacked on top of each other, which causes a further inaccuracy of cellular specificity. Consequently, the genetic information of adjacent contaminating cells is easily trapped within the measured spot (14). These issues can be limited by integrating additional gene expression profiles from scRNA-seq or other single-cell genomic approaches and subsequent predictions of location specific cell-type proportions. This complex procedure also called “deconvolution” (2) requires the application of certain algorithms [e.g. SPOTlight (17), SpatialDWLS (18), stereoscope (19), robust cell-type decomposition (14)]. After cell-type scoring, a scRNA-seq-based assignment can be calculated to predict the RNA localization. This process is called “mapping” and can be achieved by the integration of suitable algorithms, such as Harmony (20), LIGER (21), or Seurat Integration (22).

Due to the occurrence of mismatched data sets, the current integrating computational models used for deconvolution and subsequent mapping are reaching their limits. Therefore, it remains difficult to precisely determine the spatial context of cell subsets (2). The essential aspects are summarized below: First, pre-sequencing issues are caused by the fact that classical sc-RNAseq data show a tendency towards a higher sequencing depth compared to most ST-methods (2, 16). Second, a preparation of single cells from tissues can also induce artificial stress responses, that do not take place in intact tissues (23). Third, a loss of cell subsets during the enzymatic preparation of tissues can further induce mismatches and problems during deconvolution steps (24, 25). Fourth, it is possible that “clustering capture spots” may uncover cell subsets only captured by spatial barcoding (2). Thus, a precise decryption of cell-cell interactions by ST still remains an ambitious goal.

## Decrypting cellular communications *in situ*—Pushing the limits of *in situ* resolution by combining multimodal workflows

It is widely accepted that tissue-resident cells are continuously involved in short-range (< 200 μm) communication (2). This is of crucial importance for the maintenance of organ architecture and coordination of immune responses. Some monospecific receptor-ligand-interactions have already been decoded, highlighting distinct immunological programs (26–28). However, the mode of action by which cellular phenotype adoption takes place, especially within a given temporal microenvironment, is still not fully understood. In line with this challenging question, different *in situ* approaches have been established to uncover cell-cell communication. A combination of multiplexed ion beam imaging (MIBI) and ST has been used to evaluate receptor-ligand proximity or ligand-receptor-target co-expression. Based on these computed data and appropriate algorithms, it is feasible to determine distinct multi-cellular areas of communicating and non-communicating cell subsets within tissues (8). The achieved resolution of the techniques used in “marker-mapping” and creating immunological landscapes is promising [Slide-seq 10 μm; ST 50–100 μm; MIBI 800 × 800 μm (12, 29)]. Most recently, a bead-based method produced high-definition ST with resolutions nearly comparable to the size of individual cells (11). Therefore, an integration of different *ex vivo* and *in situ* techniques might be suitable to push the limits in contextualized modeling of spatial cellular communication (30) in the near future. However, *in silico* generated landscapes still remain constructed based on computed models of cell type specifications (29). This reconstruction of “highly probable signaling networks” is often based on scRNA-seq data, without exact pairing transcriptomic quantification with probability-based protein identification (30). Models capable of indexing both transcriptomes and epitopes by sequencing, such as CITE-seq, already exist (31) and will help to combine RNA-data and protein abundance in a contextual manner. However, as Alexander F. Schier has already asked: …*“Is “landscapes” even the proper analogy for multidimensional phenotypic complexity? Addressing these questions requires the multiplex in vivo measuring of dozens of transcripts over time and at single-cell resolution — a Holy Grail technology that is not yet available”*… (29).

## Computed cell-cell communication networks—First steps in decrypting physically interacting cells *in situ*

Unbiased mapping of omics to a spatial context opens a new dimension in the field of immunology (29). Given the ST-limitations described above and the multidimensionality of cell-interactions, a precise characterization of single cells *in situ* still seems to be a distant goal. To understand the immunological relevance of physically interacting cells [PICs, (32)] *in situ*, a combination of existing sequencing methods and data sets might be promising. Given the broad spectrum of cell-cell communication during homeostasis and pathological conditions, it is impossible to present one conceptual workflow of data processing, covering all cell subsets and immunological responses. Thus, I would like to address this aspect of “PIC-decryption” based on dendritic cell (DC)/T-cell interactions.

Initiation of adaptive immunity by DCs involves a cascade of fine-tuned bidirectional processes (33). We and others have been able to identify that certain subsets of DCs are mandatory for adaptive T-cell responses against pathogens (34, 35). In this context, the formation of immunological synapses between DCs and T cells is crucial for T-cell polarization (36–38). Although DC/T-cell interactions are of high clinical relevance, current genomics and imaging tools for their detection and precise *in situ* characterization are still limited, possibly due to the fact that PICs must be analyzed *in situ* on a cell-by-cell basis. There is one general problem: Within lymphoid organs, all cells are close neighbors due to the density of the tissues.

One must realize that proximity alone is not sufficient to induce cell activation or differentiation. Thus, a robust detection-signature, capable of highlighting PICs like DC/T-cell interactions by multiplex imaging systems, would be of tremendous importance for the field to understand early events in adaptive immunity (32). PICs isolated from tissues, are already under investigation (32, 39, 40). The pipeline of PIC-transcriptome analysis [abbreviated as PIC-seq (32)] is encouraging (41). One strength of this PIC-seq-concept lies in the combination of transcriptome data from *ex vivo* isolated PIC-complexes and respective single-cell data (32). If transcriptional profiles of PICs are sufficiently different, a good deconvolution is possible and PIC-seq data can be generated (42). To further calculate the transcriptional profiles of PICs, other pipelines and algorithms, such as the Giotto workflow (43), SpaOTsc algorithm (44), or CSOmap (45) might be also be implemented.

**Figure 1:**
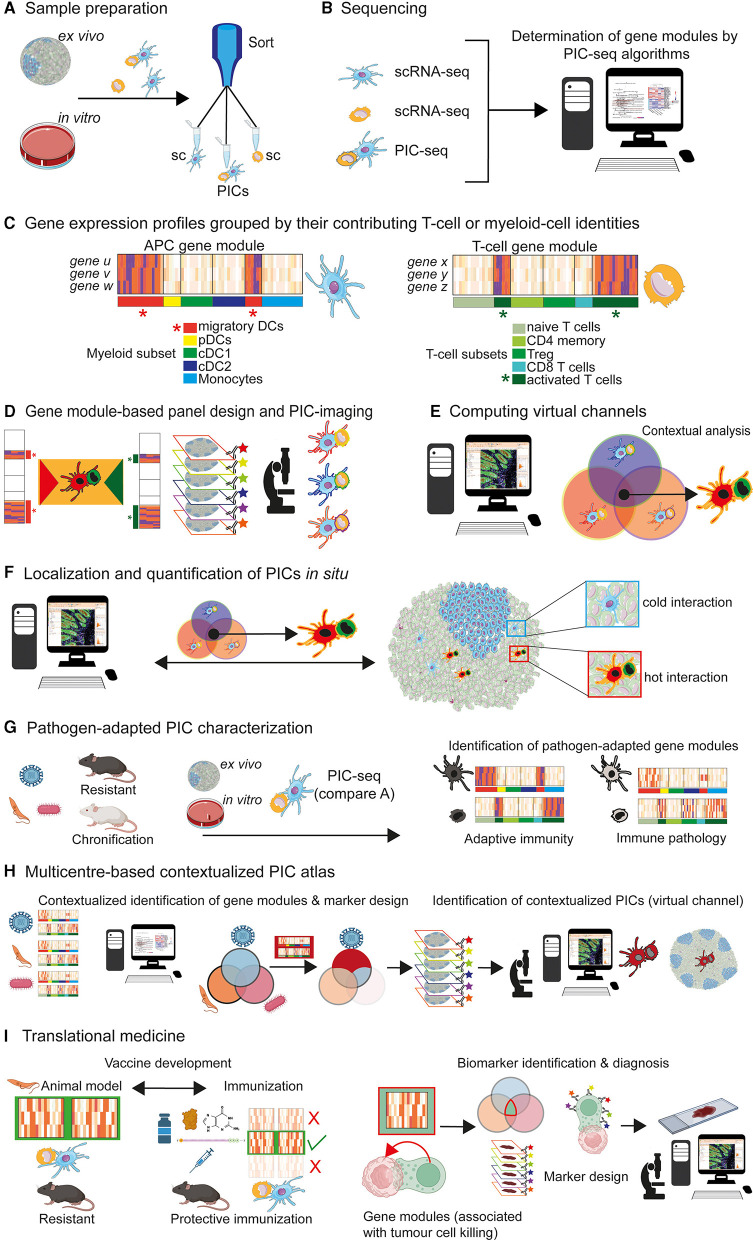
PIC characterization and implementation for contextual multiplex imaging. **(A)** *Ex vivo* and *in vitro* cultures, exposed to antigens, can be used for sorting single cells (sc; T cells or DCs) and physical interacting cells (PIC) [For further reading, see Giladi et al. (32)]. **(B)** Implementation of scRNA-seq and PIC-seq algorithms for subsequent characterization of gene modules (exemplary for T cells and DCs). **(C)** Grouped by their contributing myeloid or T-cell subset identities, it is possible to assign distinct gene modules to PICs: e.g., *genes u, v, w* to DC subsets and *genes x, y, z* to T-cell subsets [For further reading, see Giladi et al. (32)]. The putative gene expression profiles of PIC-contributing cell subsets are depicted. Specific “APC gene modules” of migratory DCs (red star) and “T-cell gene modules” of activated T cells (green star) can be generated [For further reading, see Giladi et al. (32)]. **(D)** Gene modules assigning specific cellular phenotypes can be used for panel designs and multiplex imaging. PICs consisting of migratory DCs and activated T cells can be computed (orange insert). A contextualized detection of “migratory DCs” physically interacting with “activated T cells” becomes possible (highlighted by distinct DC and T cell shapes and colors). **(E)** Based on the phenotype of cells that contribute to PICs, a virtual channel (orange contour) can be generated by imaging software tools, capable of contextualized image analysis *in situ*. PICs (orange contour) consisting of “migratory DCs (red shape)” and “activated T cells (green shape)” can be computed. **(F)** Visualization and further characterization of PICs of interest (orange contour). It is feasible to dissect “cold” cell interactions (blue insert; non-reactive cells) from “hot” cell interactions (red insert; reactive cells) resulting in T-cell activation. **(G)** Different pathogens (viruses, protozoan, bacteria etc.) and models must be considered for the generation of context-adapted gene modules. This would allow a detailed characterization of gene modules of PICs in a pathogen-specific manner. A contextualized generation of PIC-associated gene modules will permit the decryption of two central immunological categories: beneficial adaptive immune responses, resulting in protective host defense mechanisms against pathogens and immunopathological process, associated with chronic diseases. **(H)** Multicentre data acquisition and storage (PIC atlas) for the long-term generation of contextualized gene models and subsequent marker design. This concept will allow an allocation of certain immunological attributes to PICs, in a contextualized manner. A spatial detection of PICs contributing to disease chronification or successful immunity would become possible. **(I)** Conceivable applications of the PIC-seq concept in translational medicine are depicted. Left side: vaccination development. Based on experimental models and definition of gene modules (green box), it is possible to define PICs that are contributing to protective immunity against pathogens. This protective gene signatures can be compared with gene modules of PICs that occur after immunization with different vaccine protocols. Such an approach might be useful for the selection of most promising (green check) and inefficient (red cross) vaccine formulation. Right side: biomarker identification in tumour immunology. Physical interactions among tumour and immune cells are supposed to play crucial roles in immune modulation, progression and response to treatments (46). Thus, contextualized analysis of tumour-immune communications would improve the understanding of the tumour-immune interface. Comparable to the described immune cell interactions [compare **(A–H)**] a biocomputational analysis of gene modules, associated with physically interacting immune and tumour cells, might help to identify biomarkers, involved in protective tumour-immune interactions. The integration of those biomarker in high precision imaging, would lead to massive improvements of contextual resolutions regarding tumour cell/effector cell interactions. This feature will help to correlate microenvironmental neoplasia with molecular modifications—aspects that are crucial for the evaluation of tumour progression and therapy controls.

Using PIC-seq and a dermal infection model with *Nippostrongylus brasiliensis* (Nb), it could be shown that PICs consisting of dermal-derived DCs that present Nb-antigens to T cells, upregulate distinct transcriptional profiles—also called gene modules (32). In case of Nb-infection, this DC-specific gene module is composed of chemokines (Ccl22 and Ccl17) and co-stimulatory genes CD40, Ebi3, and Dll4 (32). The T cell-specific gene modules of PICs seem to be more complex, due to the heterogeneity of T cells that interact with DCs (32). However, co-culture experiments revealed that T cells that interact with antigen-presenting DCs show a reduced Th-precursor program (Klf2, Sell) associated with an induction of interferon type-I response (Stat1, Irf7), and metabolic programs (Myc and Npm1), as well as an upregulation of cytokines, chemokine receptors and effector genes (Tigit, Il22, Cxcr6, Pdcd1, and Tnfrsf9) (32). Based on these data, it can be concluded that DCs, which physically interact with T cells (Figures 1A–C), express distinct PIC-associated gene modules (32). Consequently, it is possible to design gene module-derived staining panels, allowing a refined identification of DC/T-cell interactions by multiplex imaging and tissue image cytometry (47) (Figures 1D–F).

## From PIC-associated gene modules to functionality: antibody-based multiplex imaging might represent a powerful tool for the characterization of PICs in translational clinical research

Focusing on physically interacting DC/T cells, Gialdi and colleagues could demonstrate that PICs are associated with a distinct expression of gene modules, under defined experimental conditions (32) (Figures 1A–E). This aspect represents a major limitation of the PIC-approach. Based on the huge antigen repertoire of pathogens and the corresponding heterogeneity of adaptive immunity, a context-adapted generation of gene modules is of crucial importance to avoid restrictive and oversimplified conditions. This approach is also necessary to ensure the determination of pathogen-adapted gene modules, expressed by PICs of interest (Figure 1G). Referring to the complexity of possible DC/T-cell interactions, a multicentre global database might represent a mandatory prerequisite for the identification of contextualized gene modules (Figure 1H). In line with the integration of high-dimensional data sets, an acceptable point of data reduction can be achieved, allowing the compilation of gene module-based antibody panels, useful for the spatial characterization of PICs, in a context-dependent manner (Figure 1H). This strategy might be further integrated into the new discipline of spatiotemporal molecular medicine, which aims to decrypt pathological processes within a spatial context (48, 49). A variety of applications in the field of basic research and translational medicine are conceivable. Two aspects are of particular importance in translational medicine: identification of potent vaccination strategies and biomarker identification in the field of tumour immunology (Figure 1I).

## Conclusion

It is quite clear that antigen-specific immunity represents more than the sum of its parts. Based on the multimodal incorporation of single-cell omics, ST, PIC-seq, and other cutting-edge technologies, deep-learning reconstruction of gene-regulatory and cellular networks *in situ* will become possible soon. This will be of central importance to understand the cellular crosstalk in tissues and for the decryption of complex immune responses within pathological tissues at a so far unknown level.

## Author contributions

Conceptualization, investigation, and writing—original draft: UR.
